# A space-time analysis of the proportion of late stage breast cancer in Massachusetts, 1988 to 1997

**DOI:** 10.1186/1476-072X-4-15

**Published:** 2005-06-08

**Authors:** T Joseph Sheehan, Laurie M DeChello

**Affiliations:** 1 University of Connecticut School of Medicine, Department of Community Medicine and Health Care, 263 Farmington Avenue, MC6325, Farmington, Connecticut, USA

## Abstract

**Background:**

Early detection is the best way to control breast cancer. This observational epidemiologic study uses ten years of data, 1988–1997, to determine whether the observed variations in the proportion of breast cancers diagnosed at late stage are simply random or are statistically significant with respect to both geographical location and time.

**Results:**

A total of three spatial-temporal areas were found to deviate significantly from randomness in the unadjusted analysis; one of the three areas contained statistically significant excesses in proportion of late stage, while two areas were identified as significantly lower than expected. The area of excess spanned the first three years of the study period, while the low areas spanned the last five years of the study period. Some of these areas were no longer statistically significant when adjustments were made for SES and urban/rural status.

**Conclusion:**

Although there was an area of excess in eastern Massachusetts, it only spanned the first three years of the study period. The low areas were fairly consistent, spanning the last five years of the study period.

## Background

Breast cancer is the most common cancer among women (excluding non-melanoma skin cancers). Early detection is the primary way to control breast cancer since survival drops sharply for late stage diagnoses[1] Since the proportion of late stage diagnoses in a geographic area can be viewed as a proxy for screening efficacy, this study determines whether the observed variations in the proportion of late stage cases is simply random or is statistically significant in space-time areas. A previous study looked at this same issue in Massachusetts using cases diagnosed between 1982 and 1986[2] It analyzed these data in aggregate and as a space-time model finding a single area with a significantly higher proportion of late stage cases than the rest of the state. No other studies cited in PubMed have included Massachusetts in a spatial or space-time proportion of late-stage breast cancer analysis.

The objective of this study was to examine spatially the proportion of breast cancer cases diagnosed at late stage in Massachusetts from 1988 through 1997. It is not known whether the observed variation in geographical and temporal variations in the proportion of late stage cases is random or represents statistically significant excesses. This study examines whether there is excess variation, high or low, and whether such excesses are temporary or stable, and also examines the role of socioeconomic status (SES) and urban/rural status as covariates. Several studies have shown that low SES is a risk factor for diagnosis of breast cancer at late stage [3-8] Gregorio et al. found an increased likelihood of women in low-to-moderate income census tracts in Connecticut from 1986 to 1990.[7] However, for 1990–95, this disparity in SES and late stage diagnosis was greatly decreased from the previous time period. Living in a rural area as opposed to an urban area has also been shown to be associated with higher percentages of late stage diagnosis [9-12] This study analyzed surveillance data to identify those geographic areas that warrant closer attention because of their high or low proportion of late stage breast cancer. The department of public health can use this information to assess the effectiveness of screening and other programs.

## Methods

Ten years of data from the Massachusetts Cancer Registry (MCR) included 46,666 female invasive breast cancer cases diagnosed between 1988 and 1997. This study period was chosen since the previous study of the proportion of late stage breast cancer in Massachusetts [2] studied a period prior to our study period, 1982–1986. Also, at the time the study was initiated, 1997 was the most recent data available for analysis. For space-time analyses, we wanted 10 years to study, which made the study period 1988 to 1997. It should be noted that there is a lag of several years for cancer registries to verify and clean registry data prior to it being available for analysis. For each case, the record was designed to include information on place of residence classified according to the minor civil division (town code), ZIP Code, and census tract. The record also included the age at diagnosis, date of diagnosis, race, and stage of breast cancer where stage was the historical Surveillance, Epidemiology and End Results (SEER) summary stage: local, regional, distant and unknown. Distant stage alone was considered late stage.

### Aggregation unit

Census tracts were used as the geographic aggregation unit to conduct analyses. However, 12.5% (n = 5832) of the cases diagnosed in 1988–1997 could not be assigned a reliable residential census tract because of inaccuracies or omissions in the address information provided to the MCR. In most of these cases, a mailing address had been provided and, even after extensive research, MCR staff could not assign a reliable residential address for these patients at the time of diagnosis.

Town and census tract boundaries were compared to assign the unassigned cases to tracts. For a town containing two or more census tracts, the cases missing census tracts were randomly assigned to tracts within the town based on the proportion of the town's female population each tract contributed. There were 4440 such cases, or 9.5% of all cases. The allocation of cases should therefore be free from systematic error and any error should be localized to a particular town, while the overall patterns remain correct. The proportion of late stage cases in each census tract was computed by dividing the number of late stage cases by total number of cases in that tract.

### Spatial analyses

The spatial scan statistic software SaTScan[13] was used to perform the space-time analyses. The spatial scan statistic assumes that the proportion of late stage cases follows a Bernoulli distribution. Since the Bernoulli probability model in SaTScan does not allow for covariate adjustment, the Poisson probability model was used. All incident cases of breast cancer in Massachusetts during the study period were used as the denominator for the Poisson probability model in SaTScan. The Poisson model was tested and found to be a very good estimate of the Bernoulli model, which found the same areas significant with the same risk ratios, but elevated p-values. Therefore, use of the Poisson model was more conservative than the Bernoulli model. According to the null hypothesis, the proportion of late stage cases in a particular location is equal throughout the state. Space-time analyses were performed so that the regional variations over the entire time period, 1988–1997, could be analyzed in a single model. The data were provided by MCR in one-year intervals. The data were left in one-year intervals for the space-time analyses to allow SaTScan to determine if a particular elevation or low was significant for all years, a group of consecutive years, or even just a single year. Purely spatial analyses were also performed, which do not take time into account. The maximum spatial cluster size was first set to include up to 50% of the cases, testing for both high and low excesses. Then it was set at 25% to test for high and low excesses separately and to discover smaller, more defined areas of excess and low proportion of late stage. Each area had a likelihood that was compared to the 9,999 likelihoods from the Monte Carlo trials based on a maximum cluster size that included 50% of the cases. Those areas with likelihoods within the top 500 likelihoods from the Monte Carlo trial were statistically significant at p < 0.05. The results of the 25% spatial maximum analyses are presented in the Results section. The maximum temporal cluster size was set at 90% and also includes purely spatial clusters (temporal size = 100%).

The overall SES and urban/rural status of each tract were determined and included as covariates separately and together to determine if SES or urban/rural status could account for the high or low areas by making them disappear. An SES index was created using the method of Yost et al in a principal component analysis using varimax rotation[14] When deciding on a method to measure SES, indices that included several variables was preferred in order to have a well rounded understanding of SES. Indices that used indicators available in the U.S. Census were examined. Some researchers chose only to look at one indicator from the census. [15] Yost [14] and Krieger [16] examined several indicators, therefore we evaluated their methods. Since the variables that Yost included in her studies accounted for more of the variance in a principal component analysis, her method was chosen for use in this study.[17] Two components accounted for 80% of the variance among seven economic measures obtained from the census. The first component explained 49.1% of the variance and was made up of median income, median rent, median house value, and percent with at least a high school diploma from the 1990 census[18] The first component is referred to as wealth. The second component explained 31.0% of the variance made up of the percent unemployed, percent working class, and percent below the poverty level. The second component is referred to as poverty. These principal component results were presented in an earlier study of breast cancer incidence in Massachusetts. [17] The two scores from the principal component analysis were first tested in a Poisson regression to determine the direction and statistical significance of their association with the proportion of late stage breast cancer in census tracts. They were then included in purely spatial and space-time models as covariates along with urban/rural status.

Urban/rural status was obtained from the Massachusetts Institute for Social and Economic Research (MISER) website.[19] Towns were either classified as urban or rural. Tracts within a town were assigned the same classification as those towns. Tracts that included several towns were classified as rural since all towns within a tract were also classified as rural. This urban/rural classification was included in SaTScan analyses as a covariate by itself and along with the SES scores. The use of this binary classification of urban/rural status is consistent with other published studies of breast cancer incidence[7,11,20,21]

Although race was available for most patients, the non-white cells within tracts were very small, especially by year. After evaluation of its usefulness, it was determined that race would not produce meaningful results, and was therefore not included as a covariate in this study.

Poisson regression was performed using the SES scores and urban/rural status as predictors of the number of cases diagnosed at late stage within tracts. This analysis was performed using PROC GENMOD in SAS[22] SES scores were categorized into equal quintiles. By dividing the parameter estimate of one category by another category within a SES score or urban/rural status, the percent increase or decrease of the number of cases diagnosed at late stage from one category to the next was determined.

The figures were created using Maptitude software.[23]

## Results

### Poisson regression

The Poisson regression of proportion of late stage diagnoses within each census tract on the SES and urban/rural status of each tract uncovered an increasing trend of diagnosis of late stage from the lowest category of the poverty component of 1 to the highest category, 5. The lowest poverty category has 38% fewer cases diagnosed at late stage than the highest category. Thus, the proportion of late stage cases increases with increased poverty across all five categories of poverty. The wealth component is not so clear-cut. There was not a constant increasing or decreasing trend from the lowest to the highest categories of wealth. However, the lower four categories of wealth all had higher estimates of late stage than the highest category of wealth, the variable was dichotomized where categories 1 to 4 equal 0 and category 5 equals 1. Late stage diagnosis was also affected by urban/rural status with urban tracts having about 12% more late stage diagnoses than rural tracts. Both SES scores were significant predictors of the number of cases diagnosed at late stage with p-values of 0.002 for wealth and less than 0.0001 for poverty. Urban/rural status was just significant with a p-value of 0.048. Table 1 displays the percent change of late stage diagnosis relative to the wealth and poverty SES categories of the census tracts.

**Table 1:** Wealth and poverty SES. Relative changes in the proportion of late stage breast cancer associated with 2 levels of wealth and five levels of poverty. For wealth, category 2 represents the highest level of wealth. For poverty, category 5 represents the highest level of poverty. For example, the women in the highest poverty level, 5, had a proportion of late stage 37.4% lower than those in the lowest poverty level, category 1.

	Categories Compared	% Change
Wealth SES	1–2	+17.3
Poverty SES	1–5	-37.4
	2–5	-17.4
	3–5	-16.3
	4–5	-14.3

### Purely spatial analyses

**Figure 1:**
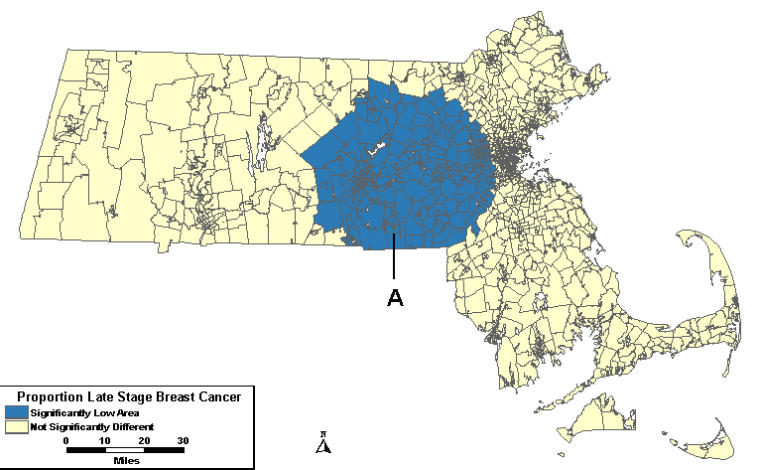
**Purely spatial, age-adjusted. **Purely spatial analysis results for age-adjusted Massachusetts proportion of late state breast cancer diagnoses, 1988–1997.

The geographic unit of analysis for all analyses was census tracts. The purely spatial scans analyzed the proportion of late stage cases with the maximum spatial window including up to 25% of the population at risk, which included all invasive breast cancer incidence diagnosed between 1988 and 1997. The observed count, relative risk (RR), and p-value for each area of excess or low proportion of late stage breast cancer can be found in Table 2 for each level of adjustment in the purely spatial analyses. The scan without covariates uncovered a large area north of Rhode Island with a statistically significantly lower proportion of late stage cases than expected. This area, Low A in Figure 1, was 19% lower than expected with 440 cases diagnosed at late stage where about 544 cases were expected.

**Table 2:** Purely spatial analysis. Proportion of late stage female breast cancer statistics for the purely spatial analyses, Massachusetts, 1988–1997. * Area not significant for this analysis.

	Low A			Low B		
	Observed	RR	p-value	Observed	RR	p-value
Not Adjusted	440	0.81	0.004	*	*	*
Adjusted for Urban/Rural Status	440	0.81	0.002	*	*	*
Adjusted for SES	*	*	*	94	0.63	0.009
Adjusted for SES & Urban/Rural Status	*	*	*	94	0.635	0.016

**Figure 2:**
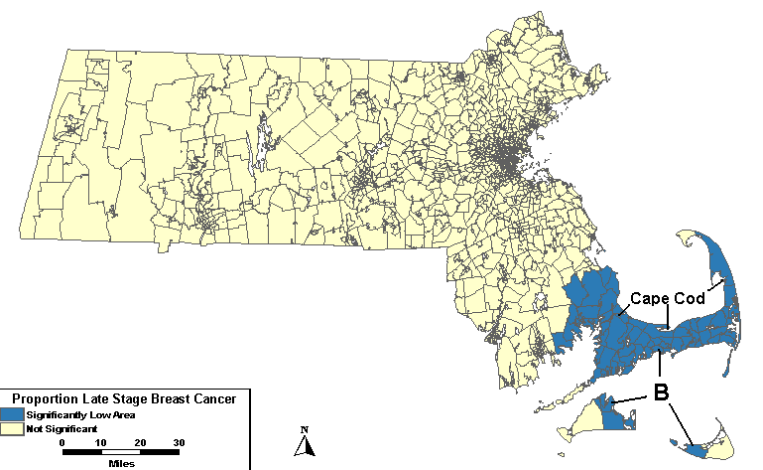
**Purely spatial, multiple adjustments. **Purely spatial analysis results for socioeconomic status- and urban/rural status-adjusted Massachusetts proportion of late state breast cancer diagnoses, 1988–1997.

The analyses were then adjusted for covariates. The analysis including urban/rural status found the same low area with no changes from the analysis without covariates. When the scan included wealth and poverty SES components as covariates, Low A was no longer significant. However, a new low area on Cape Cod and the islands, Low B, appeared with 37% fewer late stage cases than expected. This same area, Low B in Figure 2, persisted when the analysis was adjusted for both urban/rural status and SES.

### Space-time analyses

**Figure 3:**
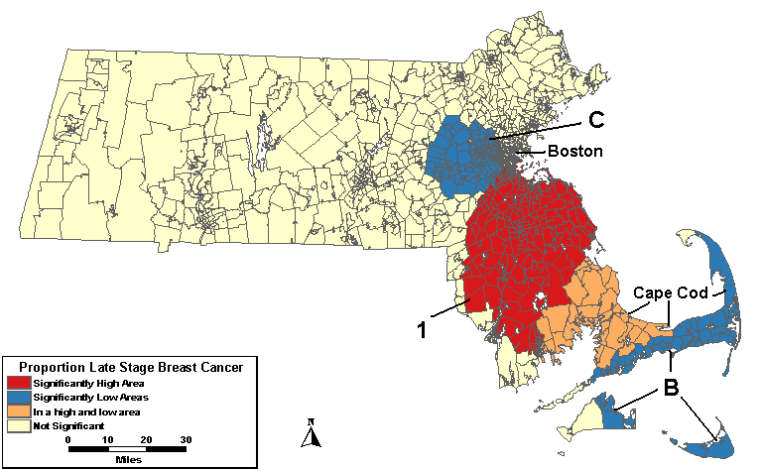
**Space-time, age-adjusted. **Space-time analysis results for age-adjusted Massachusetts proportion of late stage breast cancer diagnoses, 1988–1997.

Space-time analyses used spatial windows that could include up to 25% of the population at risk, as well as varying periods of time. The maximum temporal window of 90% was used, and included purely spatial clusters, as well. The time frame, observed count, RR and p-value for each area of excess or low proportion of late stage cancer can be found in Table 3 for each level of adjustment in the space-time analyses. The time frame in the second column of the table applies to all levels of adjustment unless otherwise noted. The analysis without covariates identified one high and 2 low areas, shown in Figure 3. High 1 was significant from 1988 to 1990 and was 52% higher than expected with 236 late stage cases when about 155 were expected. Low B covering Cape Cod and the islands was statistically significant from 1993 to 1997 with 55% fewer cases than expected. The other low area west of Boston, Low C, had 40% fewer cases with 106 late stage cases when about 178 cases were expected.

**Table 3:** Space-time analysis. Proportion of late stage female breast cancer statistics for the space-time analyses, Massachusetts, 1988–1997. ^a^Time frame for High 1 was 88–90. ^b^Time frame for Low A and Low B was 93–97. ^c^Observed count in all tracts for the area. ^d^Relative Risk Ratio. ^e^Time frame for Area 1 when adjusted for SES is 1989 alone. *Area not significant for this analysis.

	High 1^a^			Low A^b^			Low B^b^		
	Obs^c^	RR^d^	p-value	Obs^c^	RR^d^	p-value	Obs^c^	RR^d^	p-value
Not Adjusted	236	1.52	<0.0001	39	0.45	0.002	106	0.60	0.001
Adjusted for Urban/Rural Status	*	*	*	39	0.46	0.008	106	0.59	0.0003
Adjusted for SES	103^e^	1.85	0.006	39	0.42	0.0003	*	*	*
Adjusted for SES & Urban/Rural Status	103^e^	1.85	0.008	39	0.42	0.0006	*	*	*

When the analysis was adjusted for urban/rural status, High 1, seen in the analysis without covariates, was no longer significant. The lows west of Boston and on Cape Cod and the Islands remained significant. When the analyses were adjusted for wealth and poverty SES components, Low C, west of Boston, was no longer statistically significant. When the analyses were adjusted for SES and urban/rural status together, both Low B on Cape Cod and High 1 remained statistically significant. However, High 1 was only statistically significant for a single year: 1989.

## Discussion

In a previous study investigating the proportion of breast cancer cases diagnosed at late stage in Massachusetts between 1982 and 1986, a non-significant area of excess late stage was found east of Rhode Island, west of Cape Cod[2] Most of this same area was found to be statistically significantly high in the current analysis in the first three years of the study period. This area does not seem to persist as an area of significant excess after 1990, as shown in the unadjusted space-time analysis. The earlier study also found an area of significant excess in western Massachusetts. No statistically significant excesses or lows in Western Massachusetts were found in the current study.

The concentrated area of excess proportion of late stage diagnoses in Figure 3 existed only during the first 3 years of the study period. There do not appear to be any areas of significant excess proportion of late stage diagnoses in Massachusetts after 1990. The low areas identified in this study were low fro the last five of the study years.

The Poisson regression showed an inverse relationship between the number of cases diagnosed at late stage and SES. This corresponds to past published studies, which found low SES as a risk factor for late stage diagnosis of breast cancer[3-7,24]

When the space-time analyses were adjusted for urban/rural status alone, the high area found in the unadjusted analysis was no longer significant. Most of the tracts included in High 1 were classified as urban. This is contrary to previous findings where rural areas were associated with higher percentages of late stage diagnosis [9-12]

In an earlier study of breast cancer incidence in Massachusetts [17], the northwestern section of High 1 in the current study was found to have a high rate of breast cancer incidence. However, the area in the previous study was high from 1992 to 1997, which is a later time frame than the 1988 to 1990 period in the current study. Therefore, it seems as though more cases were being diagnosed and at an earlier stage in the 1990's as compared to the end of the 1980's. Eastern sections of Low A in the current study were found to have high incidence in the breast cancer incidence study of Massachusetts. [17] Although this area had a high rate of breast cancer incidence, the diagnoses are being made at an earlier stage. This seems as it might also be true for Low B on Cape Cod and western portions of Low C west of Boston.

Researchers have reported that the following factors were associated with an increased risk of late stage diagnosis of breast cancer: old age [25-27]; foreign nationality and racial and ethnic minorities [4,25,26,28,29]; living in large households, non-participation in general health check-ups, low interest in health care [25]; a body mass index greater than or equal to 25 [30]; lack of health insurance or insured by Medicaid [6,31]; low socioeconomic status [4,6,26,28,29]; and delay in seeking care [6, 32]. Arndt and colleagues also found that when tumors were detected by screening, the risk of late stage diagnosis was decreased [25]. Although data on these factors were unavailable for the current study, they might explain why certain areas have higher or lower proportions of late stage breast cancer.

MCR collects the patient's usual address of residence when diagnosed, which is in the patient's medical records; this address is used when cases are aggregated to a geographical unit. It must be noted that the address of the patient at the time of diagnosis may not be related to the cause of their cancer since this is a slowly progressing disease; the patient may have moved from the place that caused the disease. However, the place where someone is living when diagnosed might be a factor in determining if the patient is diagnosed during early or late stage of the disease. Some areas are targeted for breast cancer screening programs by public health officials. If a woman with breast cancer lives in one of those areas, she may be more likely to be diagnosed at an earlier stage than if she lived in an area where there is not an intense breast cancer screening campaign.

Since urban/rural status of census tracts was determined by the town the tracts were within or the towns that were within the tract, there is a potential weakness introduced. It is possible that towns classified as urban may have some rural sections, which may be separate tracts than the urban tract(s) of the town. The same could also be said for rural towns that may have urban tracts within the towns. However, the town level status was the smallest available geographic level with information on the urban/rural status.

There were 9.5% of the total incident cases that were randomly assigned to a census tract since they could not be geocoded to a tract by MCR as discussed in the methods section. Because of the method used to assign these cases to tracts, there should not be any systematic error and any bias would be localized within a town. Since all significant areas are larger than a town, we can conclude that these assignments did not affect the overall patterns detected.

## Conclusion

Only one area of excess late stage breast cancer diagnoses was identified in the space-time analyses for the first three years of the study period and remained statistically significant after covariate adjustment.

A low area, Low A, was found to be statistically significant in the purely spatial analyses was no longer significant after adjusting for SES. Low B on Cape Cod and the islands was not statistically significant in the purely spatial model until the model was adjusted for SES. In the space-time analysis, Low B is significant from 1993 to 1997 with or without covariate adjustment. Low C, west of Boston, was statistically significantly low for the last five years of the study period before the model was adjusted for SES.

## Authors' contributions

TJS: PI, responsible for design, funding, of project with overall responsibility for implementing the project, including the final paper. LMD: Principal data analysis, responsible for final checks on accuracy of data and all analyses, including their written interpretation.

**Figure 4:**
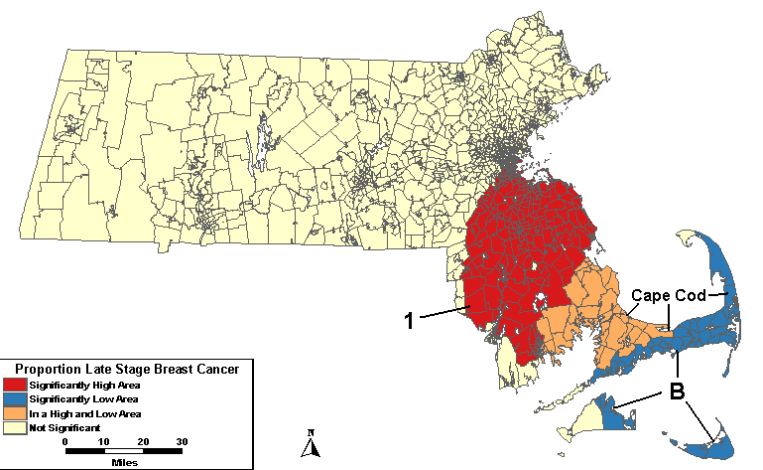
**Space-time, multiple adjustments. **Space-time analysis results for socioeconomic status- and urban/rural status-adjusted Massachusetts proportion of late stage breast cancer diagnoses, 1988–1997.
